# Synthesis and crystal structure of [Zn_6_Br_4_(C_9_H_18_NO)_4_(OH)_4_]·2C_3_H_6_O_2_

**DOI:** 10.1107/S2056989020007100

**Published:** 2020-06-02

**Authors:** Rebecca Scheel, Lukas Brieger, Kathrin Louven, Carsten Strohmann

**Affiliations:** a Inorganic Chemistry, TU Dortmund University, Otto-Hahn Str.6, 44227 Dortmund, Germany

**Keywords:** crystal structure, zinc, amino­alkoxide, zinc alkoxide, hydroxide, hydrogen bonding

## Abstract

The complete mol­ecule of the hexa­metallic title complex, namely, tetra­bromido­tetra-μ-hydroxido-hexa­kis­[μ-2-methyl-3-(pyrrolidin-1-yl)propan-2-olato]hexa­zinc(II) acetone disolvate, [Zn_6_Br_4_(C_9_H_18_NO)_4_(OH)_4_]·2C_3_H_6_O_2_, is generated by a crystallographic centre of symmetry. Two of the unique zinc atoms adopt distorted ZnO_2_NBr tetra­hedral coordination geometries and the other adopts a ZnO_3_N tetra­hedral arrangement. Both unique alkoxide ligands are *N*,*O*-chelating and both hydroxide ions are μ^2^ bridging. The crystal structure displays an O—H⋯O hydrogen bond between a μ^2^-OH group and an acetone solvent mol­ecule. The Hirshfeld surface has been calculated and is described.

## Abstract 2

The complete mol­ecule of the hexa­metallic title complex, namely, tetra­bromido­tetra-μ-hydroxido-hexa­kis­[μ-2-methyl-3-(pyrrolidin-1-yl)propan-2-olato]hexa­zinc(II) acetone disolvate, is generated by a crystallographic centre of symmetry. Two of the unique zinc atoms adopt distorted ZnO_2_NBr tetra­hedral coordination geometries and the other adopts a ZnO_3_N tetra­hedral arrangement. Both unique alkoxide ligands are *N*,*O*-chelating and both hydroxide ions are μ^2^ bridging.

## Chemical context   

Zinc complexes have a wide range of applications. For example they can be found as catalysts in organic chemistry or in the human body in enzymes, such as oxidoreductases, transferases, hydro­lases, lyases, isomerases and ligases (Lipscomb & Sträter, 1996 ▸). As a result of the filled *d* ^10^ shell of the Zn^2+^ cation, zinc complexes can exhibit different coordination geometries, including tetra­hedral, trigonal–bipyramidal and octa­hedral (Kimura *et al.*, 1997 ▸). The tetra­hedral coordination sphere is the most common because the ligands have the largest separation from each other (Holm *et al.*, 1996 ▸).

**Figure 1:**
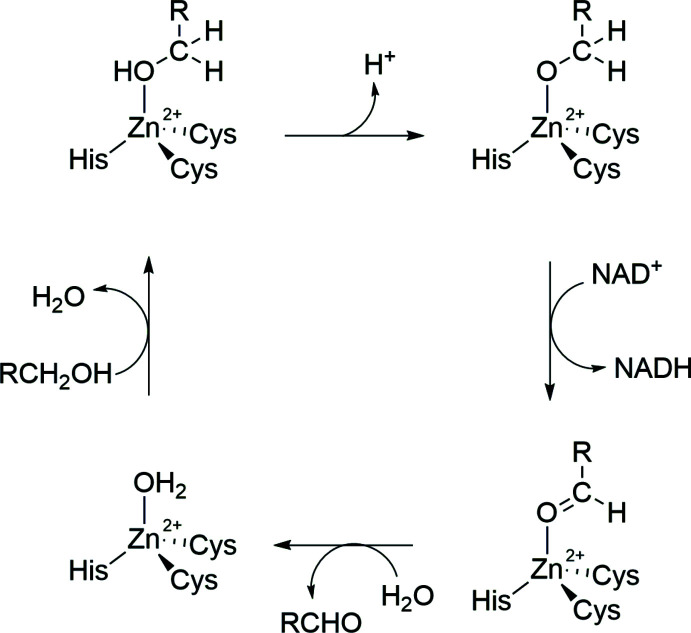
Reaction scheme of the liver alcohol de­hydrogenase cycle by the formation of a zinc alkoxide (Bergquist *et al.*, 2000 ▸).

Zinc alkoxides find applications in many fields. They are used in organic catalysis, for example in the amplification of an enanti­omer through an autocatalytic cycle by building a tetra­meric zinc alkoxide as an inter­mediate (Shibata *et al.*, 1997 ▸; Soai *et al.*, 1995 ▸). In addition, they are also used as catalysts in polymerization reactions, for example for the ring-opening polymerization of lactides (Chen *et al.*, 2006 ▸, 2011 ▸). Moreover, zinc alkoxides are electronically favoured in comparison to the incorporation of hydroxide or water mol­ecules (Bergquist & Parkin, 1999 ▸). Hence, zinc alkoxides are an important species in the human body for example for the liver alcohol de­hydrogenase or the CO_2_ transport through the circulatory system by carbonate anhydrase (Clegg *et al.*, 1988 ▸; Siek *et al.*, 2016 ▸). Liver alcohol de­hydrogenase is an enzyme that catalyses the biological oxidation of alcohols to aldehydes and ketones (Bergquist *et al.*, 2000 ▸). As part of this reaction, a tetra­hedral zinc alkoxide complex is formed and after that, a formal hydride transfer occurs from the alkoxide to the oxidized form of NAD^+^ (see Fig. 1 ▸). The entire process depicted in Fig. 1 ▸ involves the removal of a ketone from the zinc atom.

In the title compound, (I), an acetone mol­ecule inter­acts with the complex through hydrogen bonding. It can therefore be understood as an inter­mediate of the ketone removal during the de­hydrogenation process shown in Fig. 1 ▸. The remaining inter­action of the ketone with the zinc complex is inter­esting for a deeper understanding of the liver alcohol de­hydrogenase cycle.
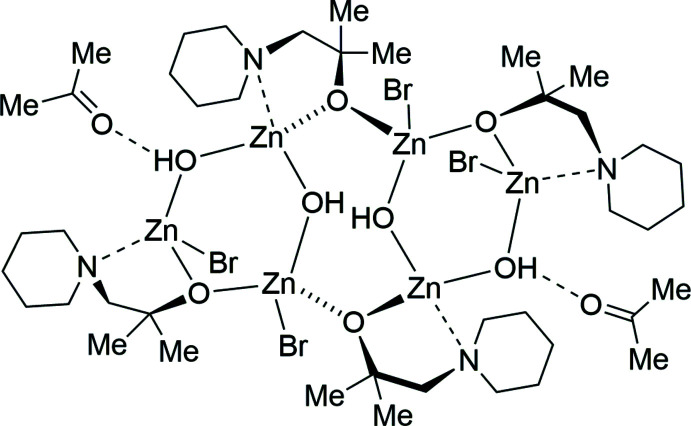


## Structural commentary   

**Figure 2:**
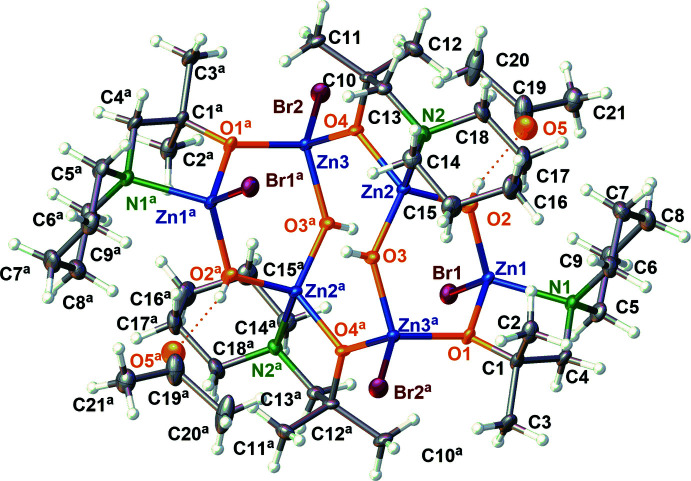
The mol­ecular structure of (I) with atom labelling and 50% displacement ellipsoids. Atoms with superscript a are generated by the symmetry operation 1 − *x*, 1 − *y*, 1 − *z*.

Compound (I) was crystallized from a mixture of zinc bromide and an amino­alkoxide in an acetone/water/tri­ethyl­amine mixture at 278 K. It crystallizes in the monoclinic crystal system in space group *P*2_1_/*n* together with one solvent mol­ecule of acetone and the complete hexa-metallic mol­ecule is generated by crystallographic inversion symmetry. The structure of (I) is shown in Fig. 2 ▸ and selected bond lengths and angles are given in Table 1 ▸.

The bond lengths between the zinc atom and the oxygen atom of the alkoxide ligand are 1.9593 (9) Å for Zn1—O1 and 1.9401 (9) Å for Zn2—O4. The bond length for Zn2 may be shorter because of the direct bonding of a bromide ion to Zn1. Bond lengths between a zinc atom and an alkoxide oxygen atom have been observed to be 1.936 (3) Å (Chen *et al.*, 2014 ▸) and 1.971 (2) Å (Siek *et al.*, 2016 ▸), thus the corresponding bonds in (I) lie between these limits. The bond lengths between the zinc atom and the bridging hydroxide O atom, Zn1—O2 and Zn2—O3, are 1.9165 (10) Å and 1.9147 (9) Å, respectively, which are elongated in comparison to a similar zinc–hydroxide bond in the literature, where the distance is 1.900 (2) Å (Siek *et al.*, 2016 ▸). However, the Zn1—Br1 [2.3816 (2) Å] and the Zn3—Br2 bonds [2.3722 (2) Å] are similar to other zinc—bromine bonds in related complexes [*e.g.* 2.358 (1) and 2.401 (1) Å; Chen *et al.*, 2014 ▸]. Finally, (I) exhibits zinc–nitro­gen bond lengths of 2.1058 (11) Å for Zn1—N1 and 2.1358 (11) Å for Zn2—N2. A similar Schiff-base complex containing zinc and hydroxide ions exhibits an zinc–imine bond length of 2.022 (4) Å (Chen *et al.*, 2014 ▸), thus the bonds in (I) are slightly elongated in comparison, especially the Zn2—N2 bond.

In general, the bond angles in (I) are as expected (Table 1 ▸), apart from the O—Zn—N angles: these are significantly compressed from the ideal tetra­hedral values with O1—Zn1—N1 = 88.54 (4)° and O4—Zn2—N2 = 86.91 (4)°, presumably because of the rigid structure of the amino­alkoxide and the higher steric demand of the tetra­hedral nitro­gen atom. This is supported by a similar compound in the literature with an O—Zn—N angle of 94.1 (1)° (Chen *et al.*, 2014 ▸). The N2—Zn2—O3 bond angle [112.11 (4)°] is slightly wider than the ideal tetra­hedral angle, as is O2—Zn2—O4 [116.23 (4)°] but O2—Zn2—O3 is slightly compressed to 108.79 (4)°. The angle of the O2 hydroxyl oxygen atom, Zn1 and the O1 atom of the alkoxide is 111.19 (4)°, which is slightly expanded from the ideal tetra­hedral angle. Finally, the N1—Zn1—Br1 bond angle is widened to 114.35 (3)°, which is similar to a compound in literature, where the corresponding angle is 113.1 (1)° (Chen *et al.*, 2014 ▸).

The central structural features of (I) are two six-membered rings, which consist of zinc–oxygen bonds (Fig. 2 ▸). In the six-membered rings two zinc atoms are bridged by one oxygen atom of the alkoxide and the other zinc centres are bridged by a hydroxide ion. Then, both six-membered rings are connected by two oxygen atoms of the alkoxide species, so the two parts are inter­connected to each other and a central eight-membered ring is formed by the connection of the two six-membered rings. The four nitro­gen atoms of the piperidine rings coordinate to the zinc atoms of the six-membered ring. The coordination spheres of the other zinc atoms are completed by bromide ions. The chelating 2-methyl-1-(piperidine-1-yl)propan-2-olate anions lie at the edges of the complex, so they do not inter­act with the other anions.

One of the methyl groups of the acetone solvent mol­ecule is disordered over two sets of sites with occupancies of 0.519 (6) and 0.481 (6). The disorder of just one methyl group of an acetone mol­ecule has already been reported in the literature (Arias *et al.*, 2013 ▸; Balogh-Hergovich *et al.*, 1998 ▸).

## Supra­molecular features   

**Figure 3:**
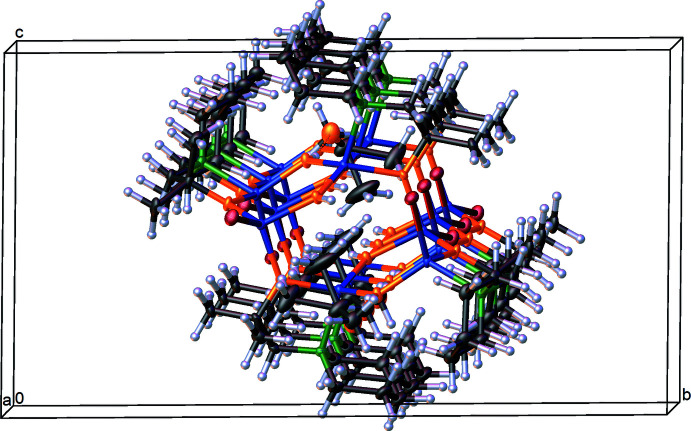
View along the *a*-axis direction of the crystal packing of (I).

In the extended structure of (I), the mol­ecules are stacked along the *a* axis, as shown in Fig. 3 ▸. As noted already, an O—H⋯O hydrogen bond links the O2—H2 hydroxide ion with the acetone solvent mol­ecule (Table 2 ▸). The graph-set motif of the O—H⋯O hydrogen bonding is described by a discrete finite pattern [*D*(2)] and, because of the inversion symmetry of the complex, a second [*D*_2_^2^(11)] pattern appears.

**Figure 4:**
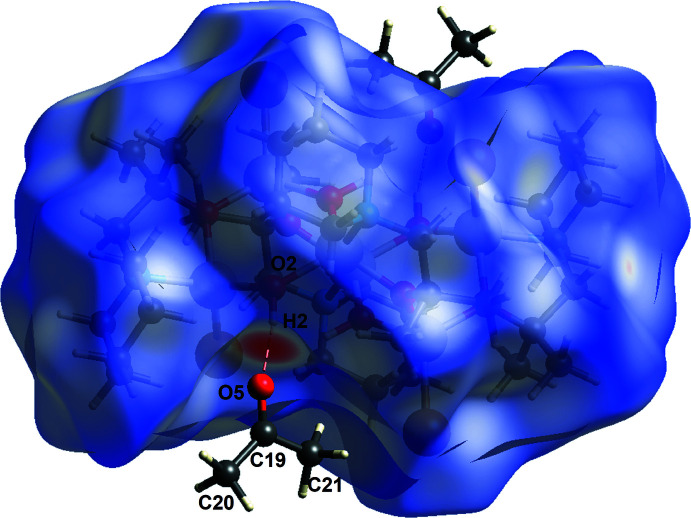
Hirshfeld surface of (I): the O—H⋯O hydrogen bond between H2 and O5 is labelled.

The Hirshfeld surface analysis of (I) (*CrystalExplorer17*; Turner *et al.*, 2017 ▸) highlights the hydrogen bonding between the main mol­ecule and the acetone solvent mol­ecule. The main mol­ecule is shown (Fig. 4 ▸) with *d*_norm_ in the range −0.5240 to +1.5598: the characteristic red spot adjacent to H2 indicates the hydrogen bond to O5. As a result of steric shielding, no inter­molecular hydrogen bonding through the bridging O3 hydroxide group occurs.

## Database survey   

Other examples of crystallographically characterized zinc complexes containing coordinated bromide ions or amino­alkoxides include Zn_2_Br_2_OH_2_(C_27_H_33_N_3_O_2_)·C_2_H_3_N [CSD (Groom *et al.*, 2016 ▸]) refcode COCQOC; Chen *et al.*, 2014 ▸] and ZnBr_2_(C_25_H_31_Cl_2_N_3_O_2_) (COCQAO; Chen *et al.*, 2014 ▸), ZnOH(C_21_H_37_BN_9_) (RUWSOT; Siek *et al.*, 2016 ▸), ZnBr(C_16_H_18_N_4_O)ZnH_2_OBr_3_·2H_2_O (SEQROY; Purkait *et al.*, 2018 ▸), ZnBr(C_21_H_22_N_6_)·ZnOCH_4_Br_3_ (MATFEV; Herber *et al.*, 2017 ▸), ZnBr(C_8_H_20_N_4_O)·ClO_4_ (BAMZAR; Reichenbach-Klinke *et al.*, 2003 ▸), ZnI_2_(C_14_H_21_BrN_2_O)·CH_4_O (DUHJIA; Zhu *et al.*, 2009 ▸), ZnCl(C_16_H_13_BrN_3_O·CH_4_O (GAVSOM; Qiu & Tong, 2005 ▸), Zn(C_2_H_5_)(C_21_H_29_BrN_3_O (FEKMIU; Stasiw *et al.*, 2017 ▸), ZnBr(C_26_H_19_N_5_O)·Br (LIMBAM; Bachmann *et al.*, 2013 ▸), ZnBr(C_15_H_18_N_3_O) (POGJAW; Ondráček *et al.*,1994 ▸), ZnBr_2_(C_10_H_24_N_2_) (DAGMUV01; Eckert *et al.*, 2013 ▸), ZnBr_2_(C_23_H_34_N_2_Si) (DASCIL; Gessner & Strohmann, 2012 ▸), Zn_2_Br_4_(C_8_H_19_NOSi)_2_ (VUPFES; Däschlein *et al.*, 2009 ▸), Zn_2_Br_2_(C_11_H_23_NO)_2_ (OMAHAM; Gessner *et al.*, 2010 ▸), Zn_2_Br_2_(C_15_H_22_FeNOSi)_2_·C_3_H_6_O (FAWPOL; Golz *et al.*, 2017 ▸) and Zn_2_Br_4_(C_18_H_23_NOSi)_2_ (VUPFAO; Däschlein & Strohmann, 2009 ▸).

## Synthesis and crystallization   

Zinc bromide (432 mg, 1.92 mmol, 3.00 eq.) was dissolved in 2.00 ml of acetone/water (*v*:*v* = 4:1). Then, 2-methyl-1-(piperidine-1-yl)propan-2-ol (200 mg, 1.28 mmol, 2.00 eq.) and tri­ethyl­amine (0.10 ml, 0.64 mmol, 1.0 eq.) were added. The reaction solution turned dull and was stored at 278 K for seven days during which time (I) crystallized as colourless blocks.

## Refinement   

Crystal data, data collection and structure refinement details are summarized in Table 3 ▸. The O-bound hydrogen atoms were located in difference-Fourier maps and refined independently. All C-bound hydrogen atoms were placed in geometrically calculated positions (C—H = 0.98–0.99 Å) and refined as riding atoms with the constraint *U*_iso_(H) = 1.5*U*_eq_(C-meth­yl) and 1.2*U*_eq_(C) for other H atoms.

## Supplementary Material

Crystal structure: contains datablock(s) I. DOI: 10.1107/S2056989020007100/hb7910sup1.cif

Structure factors: contains datablock(s) I. DOI: 10.1107/S2056989020007100/hb7910Isup2.hkl

CCDC reference: 2005919

Additional supporting information:  crystallographic information; 3D view; checkCIF report

## Figures and Tables

**Table 1:** Selected geometric parameters (Å, °)

Zn1—O1	1.9593 (9)	Zn2—O4	1.9401 (9)
Zn1—O2	1.9165 (10)	Zn2—N2	2.1358 (11)
Zn1—N1	2.1058 (11)	Zn3—O1^i^	1.9646 (9)
Zn1—Br1	2.3816 (2)	Zn3—O3^i^	1.9681 (9)
Zn2—O2	1.9310 (10)	Zn3—O4	1.9512 (9)
Zn2—O3	1.9147 (9)	Zn3—Br2	2.3722 (2)
			
O1—Zn1—Br1	114.12 (3)	O4—Zn2—N2	86.91 (4)
O1—Zn1—N1	88.54 (4)	O1^i^—Zn3—Br2	118.00 (3)
O2—Zn1—Br1	110.82 (3)	O1^i^—Zn3—O3^i^	106.38 (4)
O2—Zn1—O1	111.19 (4)	O3^i^—Zn3—Br2	111.70 (3)
O2—Zn1—N1	116.18 (4)	O4—Zn3—Br2	117.11 (3)
N1—Zn1—Br1	114.35 (3)	O4—Zn3—O1^i^	105.41 (4)
O2—Zn2—O4	116.23 (4)	O4—Zn3—O3^i^	95.52 (4)
O2—Zn2—N2	110.18 (4)	Zn1—O1—Zn3^i^	118.87 (5)
O3—Zn2—O2	108.79 (4)	Zn1—O2—Zn2	123.95 (5)
O3—Zn2—O4	120.54 (4)	Zn2—O3—Zn3^i^	133.08 (5)
O3—Zn2—N2	112.11 (4)	Zn2—O4—Zn3	120.17 (4)

Symmetry code: (i) 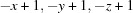.

**Table 2:** Hydrogen-bond geometry (Å, °)

*D*—H⋯*A*	*D*—H	H⋯*A*	*D*⋯*A*	*D*—H⋯*A*
O2—H2⋯O5	0.69 (2)	2.23 (2)	2.9036 (15)	166 (3)

**Table 3:** Experimental details

Crystal data	
Chemical formula	[Zn_6_Br_4_(C_9_H_18_NO)_4_(OH)_4_]·2C_3_H_6_O_2_
*M*_r_	1521.02
Crystal system, space group	Monoclinic, *P*2_1_/*n*
Temperature (K)	100
*a*, *b*, *c* (Å)	12.1464 (7), 21.0777 (12), 12.5842 (7)
β (°)	115.277 (2)
*V* (Å^3^)	2913.3 (3)
*Z*	2
Radiation type	Mo *K*α
μ (mm^−1^)	5.22
Crystal size (mm)	0.22 × 0.17 × 0.13

Data collection	
Diffractometer	Bruker D8 VENTURE area detector
Absorption correction	Multi-scan (*SADABS*; Bruker, 2016 ▸)
*T*_min_, *T*_max_	0.638, 0.746
No. of measured, independent and observed [*I* > 2σ(*I*)] reflections	87000, 10668, 9283
*R*_int_	0.044
(sin θ/λ)_max_ (Å^−1^)	0.760

Refinement	
*R*[*F* ^2^ > 2σ(*F* ^2^)], *wR*(*F* ^2^), *S*	0.021, 0.051, 1.03
No. of reflections	10668
No. of parameters	323
H-atom treatment	H atoms treated by a mixture of independent and constrained refinement
Δρ_max_, Δρ_min_ (e Å^−3^)	1.00, −1.02

Computer programs: *APEX2* and *SAINT* (Bruker, 2016 ▸), *SHELXT2014/5* (Sheldrick, 2015*a* ▸), *SHELXL2014/7* (Sheldrick, 2015*b* ▸), *OLEX2* (Dolomanov *et al.*, 2009 ▸), *Mercury* (Macrae *et al.*, 2020 ▸) and *publCIF* (Westrip, 2010 ▸).

## supplementary crystallographic information

### Crystal data

**Table:**

[Zn_6_Br_4_(C_9_H_18_NO)_4_(OH)_4_]·2C_3_H_6_O_2_	*F*(000) = 1536
*M**_r_* = 1521.02	*D*_x_ = 1.734 Mg m^−^^3^
Monoclinic, *P*2_1_/*n*	Mo *K*α radiation, λ = 0.71073 Å
*a* = 12.1464 (7) Å	Cell parameters from 9842 reflections
*b* = 21.0777 (12) Å	θ = 2.6–32.7°
*c* = 12.5842 (7) Å	µ = 5.22 mm^−^^1^
β = 115.277 (2)°	*T* = 100 K
*V* = 2913.3 (3) Å^3^	Block, colourless
*Z* = 2	0.22 × 0.17 × 0.13 mm

### Data collection

**Table:**

Bruker D8 VENTURE area detector diffractometer	10668 independent reflections
Radiation source: microfocus sealed X-ray tube, Incoatec Iµs	9283 reflections with *I* > 2σ(*I*)
HELIOS mirror optics monochromator	*R*_int_ = 0.044
Detector resolution: 10.4167 pixels mm^-1^	θ_max_ = 32.7°, θ_min_ = 2.6°
ω and φ scans	*h* = −18→18
Absorption correction: multi-scan (SADABS; Bruker, 2016)	*k* = −31→31
*T*_min_ = 0.638, *T*_max_ = 0.746	*l* = −19→19
87000 measured reflections	

### Refinement

**Table:**

Refinement on *F*^2^	Primary atom site location: dual
Least-squares matrix: full	Hydrogen site location: mixed
*R*[*F*^2^ > 2σ(*F*^2^)] = 0.021	H atoms treated by a mixture of independent and constrained refinement
*wR*(*F*^2^) = 0.051	*w* = 1/[σ^2^(*F*_o_^2^) + (0.0215*P*)^2^ + 1.5066*P*] where *P* = (*F*_o_^2^ + 2*F*_c_^2^)/3
*S* = 1.03	(Δ/σ)_max_ = 0.003
10668 reflections	Δρ_max_ = 1.00 e Å^−^^3^
323 parameters	Δρ_min_ = −1.02 e Å^−^^3^
0 restraints	

### Special details

**Table:**

Geometry. All esds (except the esd in the dihedral angle between two l.s. planes) are estimated using the full covariance matrix. The cell esds are taken into account individually in the estimation of esds in distances, angles and torsion angles; correlations between esds in cell parameters are only used when they are defined by crystal symmetry. An approximate (isotropic) treatment of cell esds is used for estimating esds involving l.s. planes.

### Fractional atomic coordinates and isotropic or equivalent isotropic displacement parameters (Å^2^)

**Table:**

	*x*	*y*	*z*	*U*_iso_*/*U*_eq_	Occ. (<1)
Zn1	0.62156 (2)	0.63111 (2)	0.41114 (2)	0.01274 (3)	
Zn2	0.45211 (2)	0.49658 (2)	0.32741 (2)	0.01142 (3)	
Zn3	0.61346 (2)	0.38070 (2)	0.51363 (2)	0.01140 (3)	
Br1	0.79563 (2)	0.61003 (2)	0.59007 (2)	0.02105 (3)	
Br2	0.80568 (2)	0.34023 (2)	0.54000 (2)	0.02140 (3)	
O1	0.48677 (8)	0.67133 (4)	0.43216 (9)	0.01519 (17)	
O2	0.56873 (9)	0.55579 (5)	0.31759 (9)	0.01716 (18)	
H2	0.616 (2)	0.5432 (11)	0.309 (2)	0.037 (6)*	
O3	0.37153 (9)	0.53555 (5)	0.41228 (9)	0.01578 (17)	
H3	0.3282 (18)	0.5134 (10)	0.4186 (17)	0.022 (5)*	
O4	0.50357 (8)	0.40863 (4)	0.35585 (8)	0.01269 (16)	
N1	0.63568 (10)	0.71351 (5)	0.32313 (10)	0.0155 (2)	
N2	0.32698 (10)	0.46745 (5)	0.15543 (9)	0.01465 (19)	
C1	0.45082 (12)	0.72981 (6)	0.36876 (13)	0.0174 (2)	
C2	0.41477 (15)	0.77873 (7)	0.43809 (15)	0.0255 (3)	
H2A	0.3434	0.7635	0.4478	0.038*	
H2B	0.3952	0.8191	0.3954	0.038*	
H2C	0.4826	0.7849	0.5156	0.038*	
C3	0.34282 (13)	0.71765 (7)	0.25016 (14)	0.0236 (3)	
H3A	0.3621	0.6823	0.2103	0.035*	
H3B	0.3264	0.7559	0.2014	0.035*	
H3C	0.2708	0.7070	0.2628	0.035*	
C4	0.56436 (13)	0.75801 (6)	0.36102 (13)	0.0190 (2)	
H4A	0.5381	0.7941	0.3054	0.023*	
H4B	0.6189	0.7751	0.4392	0.023*	
C5	0.58516 (13)	0.71191 (7)	0.19220 (13)	0.0220 (3)	
H5A	0.5839	0.7555	0.1626	0.026*	
H5B	0.5002	0.6963	0.1598	0.026*	
C6	0.65865 (15)	0.66969 (8)	0.14903 (14)	0.0261 (3)	
H6A	0.6231	0.6711	0.0621	0.031*	
H6B	0.6548	0.6253	0.1731	0.031*	
C7	0.79068 (15)	0.69111 (8)	0.19897 (16)	0.0293 (3)	
H7A	0.7959	0.7333	0.1669	0.035*	
H7B	0.8389	0.6607	0.1760	0.035*	
C8	0.84214 (13)	0.69466 (8)	0.33251 (15)	0.0250 (3)	
H8A	0.8459	0.6515	0.3648	0.030*	
H8B	0.9260	0.7118	0.3645	0.030*	
C9	0.76406 (12)	0.73670 (7)	0.37067 (14)	0.0207 (3)	
H9A	0.7990	0.7376	0.4576	0.025*	
H9B	0.7649	0.7805	0.3428	0.025*	
C10	0.46559 (11)	0.37274 (6)	0.25037 (11)	0.0139 (2)	
C11	0.45016 (13)	0.30319 (6)	0.27504 (13)	0.0202 (3)	
H11A	0.5295	0.2853	0.3269	0.030*	
H11B	0.4162	0.2795	0.2009	0.030*	
H11C	0.3949	0.3001	0.3131	0.030*	
C12	0.56244 (13)	0.37841 (7)	0.20344 (13)	0.0208 (3)	
H12A	0.5782	0.4233	0.1952	0.031*	
H12B	0.5334	0.3576	0.1267	0.031*	
H12C	0.6378	0.3580	0.2584	0.031*	
C13	0.33860 (12)	0.39724 (6)	0.16641 (11)	0.0162 (2)	
H13A	0.3171	0.3791	0.0874	0.019*	
H13B	0.2786	0.3812	0.1939	0.019*	
C14	0.19942 (12)	0.48510 (7)	0.13000 (13)	0.0202 (3)	
H14A	0.1802	0.4696	0.1944	0.024*	
H14B	0.1431	0.4641	0.0565	0.024*	
C15	0.17948 (14)	0.55629 (8)	0.11732 (14)	0.0251 (3)	
H15A	0.2309	0.5772	0.1928	0.030*	
H15B	0.0933	0.5659	0.0984	0.030*	
C16	0.21093 (16)	0.58260 (9)	0.02080 (15)	0.0313 (3)	
H16A	0.1520	0.5665	−0.0566	0.038*	
H16B	0.2056	0.6295	0.0197	0.038*	
C17	0.33921 (16)	0.56243 (8)	0.04279 (14)	0.0286 (3)	
H17A	0.3560	0.5760	−0.0242	0.034*	
H17B	0.3987	0.5837	0.1144	0.034*	
C18	0.35499 (15)	0.49096 (8)	0.05796 (13)	0.0237 (3)	
H18A	0.3004	0.4698	−0.0162	0.028*	
H18B	0.4399	0.4796	0.0745	0.028*	
O5	0.76193 (12)	0.52276 (7)	0.24976 (13)	0.0363 (3)	
C19	0.86250 (16)	0.50349 (10)	0.29097 (18)	0.0379 (4)	
C20	0.9050 (6)	0.4770 (4)	0.4156 (5)	0.082 (3)	0.519 (6)
H20A	0.9538	0.5090	0.4727	0.122*	0.519 (6)
H20B	0.9546	0.4389	0.4241	0.122*	0.519 (6)
H20C	0.8340	0.4660	0.4297	0.122*	0.519 (6)
C20A	0.8773 (4)	0.4280 (2)	0.3187 (5)	0.0537 (18)	0.481 (6)
H20D	0.8394	0.4171	0.3712	0.081*	0.481 (6)
H20E	0.9640	0.4170	0.3566	0.081*	0.481 (6)
H20F	0.8375	0.4042	0.2451	0.081*	0.481 (6)
C21	0.96197 (15)	0.52689 (8)	0.26145 (16)	0.0292 (3)	
H21A	0.9265	0.5461	0.1830	0.044*	
H21B	1.0143	0.4913	0.2625	0.044*	
H21C	1.0104	0.5586	0.3195	0.044*	

### Atomic displacement parameters (Å^2^)

**Table:**

	*U*^11^	*U*^22^	*U*^33^	*U*^12^	*U*^13^	*U*^23^
Zn1	0.01269 (6)	0.00878 (6)	0.01867 (7)	−0.00006 (5)	0.00852 (6)	0.00090 (5)
Zn2	0.01198 (6)	0.00917 (6)	0.01388 (6)	−0.00076 (5)	0.00627 (5)	−0.00150 (5)
Zn3	0.01127 (6)	0.01025 (6)	0.01389 (6)	0.00137 (5)	0.00653 (5)	0.00032 (5)
Br1	0.01778 (6)	0.02193 (7)	0.02107 (7)	0.00233 (5)	0.00602 (5)	0.00430 (5)
Br2	0.01385 (6)	0.02248 (7)	0.03002 (7)	0.00588 (5)	0.01142 (5)	0.00404 (5)
O1	0.0159 (4)	0.0097 (4)	0.0236 (5)	0.0029 (3)	0.0119 (4)	0.0046 (3)
O2	0.0166 (4)	0.0126 (4)	0.0272 (5)	−0.0020 (3)	0.0140 (4)	−0.0030 (4)
O3	0.0182 (4)	0.0130 (4)	0.0204 (5)	−0.0034 (3)	0.0122 (4)	−0.0041 (3)
O4	0.0150 (4)	0.0107 (4)	0.0117 (4)	−0.0001 (3)	0.0050 (3)	−0.0031 (3)
N1	0.0167 (5)	0.0123 (5)	0.0203 (5)	−0.0016 (4)	0.0105 (4)	0.0011 (4)
N2	0.0145 (5)	0.0171 (5)	0.0122 (4)	−0.0003 (4)	0.0056 (4)	0.0006 (4)
C1	0.0197 (6)	0.0105 (5)	0.0262 (7)	0.0036 (4)	0.0137 (5)	0.0054 (5)
C2	0.0320 (8)	0.0126 (6)	0.0411 (9)	0.0065 (5)	0.0244 (7)	0.0036 (6)
C3	0.0189 (6)	0.0239 (7)	0.0286 (7)	0.0046 (5)	0.0106 (6)	0.0094 (6)
C4	0.0231 (6)	0.0094 (5)	0.0288 (7)	0.0001 (5)	0.0154 (6)	0.0020 (5)
C5	0.0225 (6)	0.0232 (7)	0.0217 (6)	0.0010 (5)	0.0108 (5)	0.0055 (5)
C6	0.0314 (8)	0.0281 (7)	0.0243 (7)	0.0003 (6)	0.0171 (6)	0.0008 (6)
C7	0.0311 (8)	0.0314 (8)	0.0367 (9)	0.0013 (6)	0.0252 (7)	0.0057 (7)
C8	0.0192 (6)	0.0257 (7)	0.0347 (8)	−0.0005 (5)	0.0160 (6)	0.0048 (6)
C9	0.0185 (6)	0.0173 (6)	0.0287 (7)	−0.0058 (5)	0.0122 (6)	0.0005 (5)
C10	0.0155 (5)	0.0123 (5)	0.0149 (5)	−0.0023 (4)	0.0075 (5)	−0.0055 (4)
C11	0.0227 (6)	0.0120 (5)	0.0261 (7)	−0.0023 (5)	0.0106 (6)	−0.0059 (5)
C12	0.0209 (6)	0.0251 (7)	0.0212 (6)	−0.0004 (5)	0.0135 (5)	−0.0043 (5)
C13	0.0163 (5)	0.0161 (6)	0.0147 (5)	−0.0034 (4)	0.0052 (5)	−0.0048 (4)
C14	0.0137 (5)	0.0239 (7)	0.0194 (6)	0.0007 (5)	0.0036 (5)	0.0019 (5)
C15	0.0217 (6)	0.0246 (7)	0.0259 (7)	0.0072 (6)	0.0071 (6)	0.0063 (6)
C16	0.0347 (8)	0.0290 (8)	0.0239 (7)	0.0058 (7)	0.0066 (7)	0.0116 (6)
C17	0.0346 (8)	0.0301 (8)	0.0234 (7)	0.0021 (7)	0.0146 (7)	0.0112 (6)
C18	0.0291 (7)	0.0294 (7)	0.0159 (6)	0.0018 (6)	0.0127 (6)	0.0040 (5)
O5	0.0309 (6)	0.0365 (7)	0.0516 (8)	0.0045 (5)	0.0273 (6)	0.0029 (6)
C19	0.0260 (8)	0.0477 (11)	0.0405 (10)	0.0031 (7)	0.0146 (7)	0.0224 (8)
C20	0.074 (4)	0.127 (6)	0.061 (3)	0.050 (4)	0.045 (3)	0.063 (4)
C20A	0.0223 (17)	0.052 (3)	0.084 (4)	0.0048 (17)	0.020 (2)	0.041 (3)
C21	0.0239 (7)	0.0305 (8)	0.0350 (8)	−0.0013 (6)	0.0143 (7)	0.0054 (7)

### Geometric parameters (Å, º)

**Table:**

Zn1—O1	1.9593 (9)	C8—H8A	0.9900
Zn1—O2	1.9165 (10)	C8—H8B	0.9900
Zn1—N1	2.1058 (11)	C8—C9	1.518 (2)
Zn1—Br1	2.3816 (2)	C9—H9A	0.9900
Zn2—O2	1.9310 (10)	C9—H9B	0.9900
Zn2—O3	1.9147 (9)	C10—C11	1.5265 (19)
Zn2—O4	1.9401 (9)	C10—C12	1.5297 (18)
Zn2—N2	2.1358 (11)	C10—C13	1.5396 (18)
Zn3—O1^i^	1.9646 (9)	C11—H11A	0.9800
Zn3—O3^i^	1.9681 (9)	C11—H11B	0.9800
Zn3—O4	1.9512 (9)	C11—H11C	0.9800
Zn3—Br2	2.3722 (2)	C12—H12A	0.9800
O1—Zn3^i^	1.9646 (9)	C12—H12B	0.9800
O1—C1	1.4314 (15)	C12—H12C	0.9800
O2—H2	0.69 (2)	C13—H13A	0.9900
O3—Zn3^i^	1.9682 (9)	C13—H13B	0.9900
O3—H3	0.73 (2)	C14—H14A	0.9900
O4—C10	1.4227 (15)	C14—H14B	0.9900
N1—C4	1.4867 (17)	C14—C15	1.517 (2)
N1—C5	1.4930 (19)	C15—H15A	0.9900
N1—C9	1.4940 (17)	C15—H15B	0.9900
N2—C13	1.4876 (17)	C15—C16	1.525 (2)
N2—C14	1.4892 (17)	C16—H16A	0.9900
N2—C18	1.4907 (17)	C16—H16B	0.9900
C1—C2	1.531 (2)	C16—C17	1.522 (2)
C1—C3	1.530 (2)	C17—H17A	0.9900
C1—C4	1.5425 (19)	C17—H17B	0.9900
C2—H2A	0.9800	C17—C18	1.520 (2)
C2—H2B	0.9800	C18—H18A	0.9900
C2—H2C	0.9800	C18—H18B	0.9900
C3—H3A	0.9800	O5—C19	1.177 (2)
C3—H3B	0.9800	C19—C20	1.532 (5)
C3—H3C	0.9800	C19—C20A	1.623 (5)
C4—H4A	0.9900	C19—C21	1.491 (2)
C4—H4B	0.9900	C20—H20A	0.9800
C5—H5A	0.9900	C20—H20B	0.9800
C5—H5B	0.9900	C20—H20C	0.9800
C5—C6	1.516 (2)	C20A—H20D	0.9800
C6—H6A	0.9900	C20A—H20E	0.9800
C6—H6B	0.9900	C20A—H20F	0.9800
C6—C7	1.520 (2)	C21—H21A	0.9800
C7—H7A	0.9900	C21—H21B	0.9800
C7—H7B	0.9900	C21—H21C	0.9800
C7—C8	1.524 (2)		
			
O1—Zn1—Br1	114.12 (3)	C9—C8—C7	111.10 (13)
O1—Zn1—N1	88.54 (4)	C9—C8—H8A	109.4
O2—Zn1—Br1	110.82 (3)	C9—C8—H8B	109.4
O2—Zn1—O1	111.19 (4)	N1—C9—C8	111.64 (12)
O2—Zn1—N1	116.18 (4)	N1—C9—H9A	109.3
N1—Zn1—Br1	114.35 (3)	N1—C9—H9B	109.3
O2—Zn2—O4	116.23 (4)	C8—C9—H9A	109.3
O2—Zn2—N2	110.18 (4)	C8—C9—H9B	109.3
O3—Zn2—O2	108.79 (4)	H9A—C9—H9B	108.0
O3—Zn2—O4	120.54 (4)	O4—C10—C11	109.87 (11)
O3—Zn2—N2	112.11 (4)	O4—C10—C12	108.80 (10)
O4—Zn2—N2	86.91 (4)	O4—C10—C13	107.02 (10)
O1^i^—Zn3—Br2	118.00 (3)	C11—C10—C12	109.57 (11)
O1^i^—Zn3—O3^i^	106.38 (4)	C11—C10—C13	106.71 (10)
O3^i^—Zn3—Br2	111.70 (3)	C12—C10—C13	114.78 (11)
O4—Zn3—Br2	117.11 (3)	C10—C11—H11A	109.5
O4—Zn3—O1^i^	105.41 (4)	C10—C11—H11B	109.5
O4—Zn3—O3^i^	95.52 (4)	C10—C11—H11C	109.5
Zn1—O1—Zn3^i^	118.87 (5)	H11A—C11—H11B	109.5
Zn1—O2—Zn2	123.95 (5)	H11A—C11—H11C	109.5
Zn2—O3—Zn3^i^	133.08 (5)	H11B—C11—H11C	109.5
Zn2—O4—Zn3	120.17 (4)	C10—C12—H12A	109.5
C1—O1—Zn1	111.77 (7)	C10—C12—H12B	109.5
C1—O1—Zn3^i^	125.97 (8)	C10—C12—H12C	109.5
Zn1—O2—H2	109 (2)	H12A—C12—H12B	109.5
Zn2—O2—H2	117 (2)	H12A—C12—H12C	109.5
Zn2—O3—H3	110.4 (15)	H12B—C12—H12C	109.5
Zn3^i^—O3—H3	116.5 (15)	N2—C13—C10	115.14 (10)
C10—O4—Zn2	112.67 (7)	N2—C13—H13A	108.5
C10—O4—Zn3	126.68 (8)	N2—C13—H13B	108.5
C4—N1—Zn1	99.33 (7)	C10—C13—H13A	108.5
C4—N1—C5	110.31 (11)	C10—C13—H13B	108.5
C4—N1—C9	108.46 (11)	H13A—C13—H13B	107.5
C5—N1—Zn1	118.32 (9)	N2—C14—H14A	109.2
C5—N1—C9	108.44 (11)	N2—C14—H14B	109.2
C9—N1—Zn1	111.39 (8)	N2—C14—C15	111.98 (12)
C13—N2—Zn2	101.16 (8)	H14A—C14—H14B	107.9
C13—N2—C14	108.49 (10)	C15—C14—H14A	109.2
C13—N2—C18	111.17 (11)	C15—C14—H14B	109.2
C14—N2—Zn2	111.77 (8)	C14—C15—H15A	109.4
C14—N2—C18	108.80 (11)	C14—C15—H15B	109.4
C18—N2—Zn2	115.13 (9)	C14—C15—C16	111.13 (13)
O1—C1—C2	110.84 (11)	H15A—C15—H15B	108.0
O1—C1—C3	109.26 (11)	C16—C15—H15A	109.4
O1—C1—C4	107.39 (10)	C16—C15—H15B	109.4
C2—C1—C4	104.92 (11)	C15—C16—H16A	109.7
C3—C1—C2	109.51 (12)	C15—C16—H16B	109.7
C3—C1—C4	114.85 (12)	H16A—C16—H16B	108.2
C1—C2—H2A	109.5	C17—C16—C15	109.80 (13)
C1—C2—H2B	109.5	C17—C16—H16A	109.7
C1—C2—H2C	109.5	C17—C16—H16B	109.7
H2A—C2—H2B	109.5	C16—C17—H17A	109.4
H2A—C2—H2C	109.5	C16—C17—H17B	109.4
H2B—C2—H2C	109.5	H17A—C17—H17B	108.0
C1—C3—H3A	109.5	C18—C17—C16	111.34 (14)
C1—C3—H3B	109.5	C18—C17—H17A	109.4
C1—C3—H3C	109.5	C18—C17—H17B	109.4
H3A—C3—H3B	109.5	N2—C18—C17	111.83 (12)
H3A—C3—H3C	109.5	N2—C18—H18A	109.3
H3B—C3—H3C	109.5	N2—C18—H18B	109.3
N1—C4—C1	115.86 (11)	C17—C18—H18A	109.3
N1—C4—H4A	108.3	C17—C18—H18B	109.3
N1—C4—H4B	108.3	H18A—C18—H18B	107.9
C1—C4—H4A	108.3	O5—C19—C20	114.2 (2)
C1—C4—H4B	108.3	O5—C19—C20A	115.6 (2)
H4A—C4—H4B	107.4	O5—C19—C21	125.10 (17)
N1—C5—H5A	109.1	C21—C19—C20	114.9 (3)
N1—C5—H5B	109.1	C21—C19—C20A	110.5 (2)
N1—C5—C6	112.41 (12)	C19—C20—H20A	109.5
H5A—C5—H5B	107.9	C19—C20—H20B	109.5
C6—C5—H5A	109.1	C19—C20—H20C	109.5
C6—C5—H5B	109.1	H20A—C20—H20B	109.5
C5—C6—H6A	109.5	H20A—C20—H20C	109.5
C5—C6—H6B	109.5	H20B—C20—H20C	109.5
C5—C6—C7	110.88 (14)	C19—C20A—H20D	109.5
H6A—C6—H6B	108.1	C19—C20A—H20E	109.5
C7—C6—H6A	109.5	C19—C20A—H20F	109.5
C7—C6—H6B	109.5	H20D—C20A—H20E	109.5
C6—C7—H7A	109.8	H20D—C20A—H20F	109.5
C6—C7—H7B	109.8	H20E—C20A—H20F	109.5
C6—C7—C8	109.51 (12)	C19—C21—H21A	109.5
H7A—C7—H7B	108.2	C19—C21—H21B	109.5
C8—C7—H7A	109.8	C19—C21—H21C	109.5
C8—C7—H7B	109.8	H21A—C21—H21B	109.5
C7—C8—H8A	109.4	H21A—C21—H21C	109.5
C7—C8—H8B	109.4	H21B—C21—H21C	109.5
H8A—C8—H8B	108.0		
			
Zn1—O1—C1—C2	−143.25 (10)	C2—C1—C4—N1	165.89 (12)
Zn1—O1—C1—C3	95.97 (10)	C3—C1—C4—N1	−73.82 (15)
Zn1—O1—C1—C4	−29.18 (13)	C4—N1—C5—C6	177.10 (12)
Zn1—N1—C4—C1	−38.23 (13)	C4—N1—C9—C8	−178.28 (12)
Zn1—N1—C5—C6	−69.58 (14)	C5—N1—C4—C1	86.76 (14)
Zn1—N1—C9—C8	73.40 (13)	C5—N1—C9—C8	−58.48 (15)
Zn2—O4—C10—C11	−152.00 (8)	C5—C6—C7—C8	54.13 (18)
Zn2—O4—C10—C12	88.05 (11)	C6—C7—C8—C9	−54.74 (17)
Zn2—O4—C10—C13	−36.51 (11)	C7—C8—C9—N1	58.21 (16)
Zn2—N2—C13—C10	−32.11 (11)	C9—N1—C4—C1	−154.62 (12)
Zn2—N2—C14—C15	69.34 (13)	C9—N1—C5—C6	58.46 (15)
Zn2—N2—C18—C17	−67.74 (14)	C11—C10—C13—N2	164.92 (11)
Zn3^i^—O1—C1—C2	57.94 (15)	C12—C10—C13—N2	−73.49 (14)
Zn3^i^—O1—C1—C3	−62.83 (13)	C13—N2—C14—C15	−179.96 (11)
Zn3^i^—O1—C1—C4	172.01 (8)	C13—N2—C18—C17	178.00 (12)
Zn3—O4—C10—C11	36.06 (13)	C14—N2—C13—C10	−149.80 (11)
Zn3—O4—C10—C12	−83.89 (12)	C14—N2—C18—C17	58.59 (16)
Zn3—O4—C10—C13	151.55 (8)	C14—C15—C16—C17	−53.46 (18)
O1—C1—C4—N1	47.91 (16)	C15—C16—C17—C18	53.38 (19)
O4—C10—C13—N2	47.33 (14)	C16—C17—C18—N2	−57.19 (17)
N1—C5—C6—C7	−57.60 (16)	C18—N2—C13—C10	90.61 (13)
N2—C14—C15—C16	57.53 (16)	C18—N2—C14—C15	−58.90 (15)

Symmetry code: (i) −*x*+1, −*y*+1, −*z*+1.

### Hydrogen-bond geometry (Å, º)

**Table:**

*D*—H···*A*	*D*—H	H···*A*	*D*···*A*	*D*—H···*A*
O2—H2···O5	0.69 (2)	2.23 (2)	2.9036 (15)	166 (3)
